# Effects of *Sophora japonica *flowers (*Huaihua*) on cerebral infarction

**DOI:** 10.1186/1749-8546-5-34

**Published:** 2010-09-27

**Authors:** Hsiang-Ni Chen, Ching-Liang Hsieh

**Affiliations:** 1 Department of Chinese Medicine, China Medical University Hospital, Taichung 40402, Taiwan; 2 Graduate Institute of Acupuncture Science, China Medical University, Taichung 40402, Taiwan; 3 Acupuncture Research Center, China Medical University, Taichung 40402, Taiwan

## Abstract

The dried flowers and buds of *Sophora japonica *are used as a medicinal herb in China, Japan and Korea to treat bleeding hemorrhoids and hematemesis. This article presents an overview of the effects of *Sophora japonica *on cerebral infarction based on literature searched from Medline, PubMed, Cochrane Library and the China National Knowledge Infrastructure (CNKI). *Sophora japonica *contains both anti-hemorrhagic and anti-hemostatic substances. *Sophora japonica *reduces cerebral infarction partly as a result of its anti-oxidative and anti-inflammatory activities. Previous studies found that *Sophora japonica *reduced the size of cerebral infarction and neurological deficits and reduced microglial activation, interleukin-1β release and number of apoptotic cells in ischemia-reperfusion injured Sprague-Dawley rats. Further study is required to determine the relationship between *Sophora japonica*-mediated reduction in cerebral infarction size and the effects of *Sophora japonica *on platelet aggregation and cardiovascular function.

## Background

Dried flowers and buds of *Sophora japonica *(*Huaihua*) are a medicinal herb used in China, Japan and Korea to treat hemorrhoids and hematemesis [1]. Main components of *Sophora japonica *include flavones, tetraglycosides, isoflavones, isoflavone tetraglycosides, triterpene glycosides, phospholipids, alkaloids, amino acids and polysaccharides [2]. Moreover, *Sophora japonica *contains five main flavonoids of rutin, quercetin, isorhamnetin, isorhamnetin, genistein and kaempferol [2].

This article aims to provide an overview of the pharmacology of *Sophora japonica*, in particular its effects and mechanisms in reducing cerebral infarction (Table 1). To this end, we searched the English language databases namely Medline, PubMed, Cochrane Library and Chinese language database namely China National Knowledge Infrastructure (CNKI) between 1980 and 2009, using *Sophora japonica *(OR quercetin OR rutin) AND cerebral infarction as the English keywords and *Huaihua *as the Chinese one. The initial search generated 173 articles from the English language databases and 141 articles from the Chinese one.

**Table 1:** Possible pharmacological actions of *Sophora japonica *on cerebral infarction

Pharmacological actions	Related components	Possible mechanisms
**Anti-oxidative effects**	flavonoid	scavenges superoxide anion and free radicals [8]
	polysaccharides	scavenges hydroxyl and superoxide anion free radicals [9]
	quercetin and rutin	scavenge free radicals [10]
	rutin	inhibits the conversion of superoxide anion to hydroxyl radicals[11]
	irisolidone	inhibits lipid peroxidation, DNA damage, and reduced apoptosis induced by hydrogen peroxide[12]
		
**Anti-inflammatory effects**	quercetin and rutin	inhibit microglial activation, interleukin-1β (IL-1β) release, and apoptosis [25]
	rutin	reduce IL-1β mRNA expression [26]
		
**Anti-platelet aggregation effects**	biochanin A, irisolidone, genistein and tectoridin	inhibit arachidonic acid- and thromboxane A_2_- induced platelet aggregation [2]
	quercetin	inhibits free calcium increase within platelets [27]
		
**Cardiovascular effects**	*Sophora japonica *decoction	reduces cardiac muscle contractility and reduces heart rate [30,31]
	Quercetin	stabilizes capillary integrity [32]
	rutin, quercetin and tannin	reduce capillary perameability [31,33]
	Isorhamnetin	increases capillary permeability [1]

### Pharmacology

#### Anti-oxidative effects

Our previous studies found that (1) levels of superoxide anion in arterial blood increased at the start of 2 hours of reperfusion after ischemia in rats with middle cerebral artery occlusion (MCAo) [3]; (2) levels of superoxide anion in the region of brain parenchymal damage increased during 2 hours of reperfusion after cerebral ischemia in ischemia-reperfusion injured rats [4]; (3) *Guizhifuling Wan *(consisting of Cinnamon twig, *Prunus persica *[L] Batsch, *Poria cocos *[Schw.] Wolf, *Paeonia lactiflora *Pall, *Paeonia suffruticosa *Andr, *Angelica sinensis *[*Oliv*. Diels and *Ligusticum chuanxiong *Hort.] with ferulic acid and *Paeonia suffruticosa *with paeonol as components and the root of *Paeonia lactflora *Pall) reduced superoxide anion levels during 2 hours of reperfusion after ischemia, cerebral infarction size and neurological deficit score in rats with MCAo [3-5]. Free radical scavenging activity was demonstrated in *Ginkgo biloba *L. leaves which contain quercetin and rutin which have free radical scavenging activity [6]. Extracts of *Ginkgo biloba *L. leaves (Egb 761) reduced the size of cerebral infarction and improved neurological behavior in rats with permanent and transient MCAo [7]. Ma *et al*. found that the flavonoid components of *Sophora japonica *scavenged superoxide anion and 1,1-diphenyl-2-picrylhydrazyl (DPPH) free radicals [8]. Simple and orthogonal experiments to study the antioxidative activites of *Sophora japonica*, Wang *et al*. found that the polysaccharides of *Sophora japonica *scavenged hydroxyl and superoxide anion free radicals [9]. Both quercetin and rutin demonstrated free radical scavenging activity; however, quercetin offered better protection. A study reported that quercetin (better than rutin) protected rabbit erythrocytes with normal and high cholesterol content against lipid peroxidation by *t*-BuOOH-induced reactive oxygen species [10]. Afanas'ev *et al*. found that rutin in iron-rutin complex inhibited the conversion of superoxide anion to hydroxyl radicals in normal and iron-overloaded rats [11]. Kang *et al*. showed that the anti-oxidant irisolidone prevented lipid peroxidation, DNA damage and reduced apoptosis induced by hydrogen peroxide in Chinese hamster lung fibroblasts [12].

Generation of reactive oxygen species (ROS) including nitrogen and oxygen free radicals played a critical role in brain damage during the reperfusion period after ischemia [13,14]. Brain damage due to ROS-mediated injury involved metabolic signal transmission among mitochondria, DNA repair enzymes and transcription factors leading to apoptosis [15]. Anti-oxidant components such as those in *Gingko biloba *extracts played a neuroprotective role [16,17]. Both quercetin and rutin scavenged free radicals, improved spatial memory and reduced neuronal death induced by repeated cerebral ischemia [18]. Rutin reduced ischemia-reperfusion injury by scavenging reactive species [14,19].

#### Anti-inflammatory effects

Microglia was activated by various types of brain damage such as ischemic and inflammatory damage [20]. This activation indicated the severity of neuronal damage in rats with MCAo [21]. The levels of IL-1β, a pro-inflammatory cytokine which is expressed mainly by glial cells such as astrocytes, oligodendrocytes and microglia [22] increased after permanent focal cerebral ischemia [23] whereas they reduced leucocyte infiltration, thereby causing neuronal damage and brain edema [24]. Similarly, our previous studies found that both *Guizhifuling Wan *and paeonol inhibited microglial activation and IL-1β release and reduced cerebral infarction size in rats with MCAo [3,5]. *Sophora japonica *inhibited microglial activation, interleukin-1β (IL-1β) release and apoptosis in rats with transient MCAo, suggesting that *Sophora japonica *reduced inflammation and prevented neuronal death by inhibiting microglial release of IL-1β, a pro-inflammatory cytokine [25]. Koda *et al*. reported that dietary rutin supplementation 10 or 20 days after trimethyltin (TMT) administration in rats reduced IL-1β mRNA expression in the microglia of the hippocampus, suggesting that rutin was neuro-protective against TMT-induced neuronal damage [26].

#### Anti-platelet aggregation effects

Kim and Yun-Chol reported that, compared with acetylsalicylic acid, biochanin A, irisolidone, genistein and tectoridin of *Sophora japonica *were stronger inhibitors of arachidonic acid- and thromboxane A_2_-induced platelet aggregation in platelet-rich and platelet-poor plasma in rats [2]. A study found that quercetin of *Sophora japonica *inhibited free calcium accumulation within platelets thereby preventing platelet aggregation [27]. Antiplatelet agents such as aspirin were recommended for the treatment of acute ischemic stroke [28]. Platelet adhesion was enhanced after MCAo and reperfusion in mice [29].

#### Effects on cardiovascular system and blood coagulation

Administration of *Sophora japonica *decoction into the jugular veins of rabbits decreased cardiac muscle contractility and reduced heart rate, suggesting that *Sophora japonica *reduced the consumption of oxygen to protect cardiac function [30,31]. The anti-hemorrhagic effect of quercetin (water extracts of the buds of *Sophora japonica*) was due to stabilization of capillary integrity [32]. Oral administration of *Sophora japonica *extracts (containing rutin, quercetin and tannin) for five days reduced capillary perameability, bleeding time and coagulation time in mice and reduced prothrombin time in rats, thereby demonstrating the hemostatic effect of *Sophora japonica *[30,33]. Isorhamnetin from *Sophora japonica *was also anti-hemostatic, as a result from increased capillary permeability and reduced platelet aggregation [1].

## Conclusion

*Sophora japonica *contains both anti-hemorrhagic and anti-hemostatic substances. *Sophora japonica *reduces cerebral infarction partly as a result of its anti-oxidative and anti-inflammatory activities. Further study is required to determine the relationship between *Sophora japonica*-mediated reduction in cerebral infarction size and its effects on platelet aggregation and cardiovascular function.

## Abbreviations

MCAO: middle cerebral artery occlusion; EGB 761: extract of Ginkgo biloba L. leaves; DPPH: 1,1-diphenyl-2-picrylhydrazyl; T-BUOOH: tert-butyl hydroperoxide; IL-1β: interleukin-1β; TMT: trimethyltin; MRNA: messenger ribonucleic acid; ROS: reactive oxygen species; DNA: deoxyribonucleic acid

## Competing interests

The authors declare that they have no competing interests.

## Authors' contributions

CLH designed the study and revised the manuscript. HNC conducted the literature search and drafted the manuscript. Both authors read and approved the final version of the manuscript.
